# MINSTED tracking of single biomolecules

**DOI:** 10.1038/s41592-024-02209-6

**Published:** 2024-03-13

**Authors:** Lukas Scheiderer, Henrik von der Emde, Mira Hesselink, Michael Weber, Stefan W. Hell

**Affiliations:** 1 Department of Optical Nanoscopy, Max Planck Institute for Medical Research, Heidelberg, Germany; 2 Department of NanoBiophotonics, Max Planck Institute for Multidisciplinary Sciences, Göttingen, Germany

**Keywords:** Super-resolution microscopy, Single-molecule biophysics

## Abstract

Here we show that MINSTED localization, a method whereby the position of a fluorophore is identified with precisely controlled beams of a STED microscope, tracks fluorophores and hence labeled biomolecules with nanometer/millisecond spatiotemporal precision. By updating the position for each detected photon, MINSTED recognizes fluorophore steps of 16 nm within <250 μs using about 13 photons. The power of MINSTED tracking is demonstrated by resolving the stepping of the motor protein kinesin-1 walking on microtubules and switching protofilaments.

## Abstract (web summary)

MINSTED quantifies tiny movements of individual biomolecules with high spatiotemporal precision to successfully resolve the steps of the molecular motor protein kinesin-1 labeled with a single fluorophore as it switches protofilaments.

## Main

Measuring conformational changes and movements of individual proteins and other biomolecules is key to understanding their function. A powerful approach to this end is labeling the biomolecule with a fluorophore and tracking its position with an optical microscope. In contrast to scattering gold or latex beads, fluorophores are much smaller than proteins and can be specifically linked to numerous protein sites with minimal functional interference. However, their relatively low photon rates limit the spatiotemporal resolution obtainable in camera^1–3^ or confocal-microscopy-based^4^ tracking, because the localization precision of these techniques scales with $1/\sqrt{N}$, with *N* denoting the number of detected photons. A way out of this catch is offered by localization through optical coordinate-targeting as realized in the methods called MINFLUX^5,6^ and MINSTED^7^. These methods typically harness a donut-shaped laser beam having a central intensity minimum in order to optically target a reference coordinate in the focal plane with nearly infinite precision. Localization then boils down to finding out the unknown position of the fluorophore relative to the position targeted by the donut minimum. Ideally, coordinate-targeted localization is performed iteratively, by continually relocating the donut minimum such that the average distance between the minimum and the fluorophore becomes smaller in each iteration. As a result, the localization precision scales with e^−*N*^ (refs. ^8,9^), rather than slowly with $1/\sqrt{N}$, making coordinate-targeted localization with a given *N* more photon efficient. In MINFLUX, the wavelength of the laser donut beam excites fluorophores. Therefore, the fluorophore is at the donut position where its fluorescence rate would be minimal. In contrast, MINSTED uses a regularly focused beam for excitation and the donut is used for de-excitation through stimulated emission depletion (STED)^10^. Hence, in localization by MINSTED the fluorophore is at the donut position where the fluorescence rate would be maximal. Yet despite this marked difference, both MINFLUX and MINSTED attained Angström localization precision in superresolution imaging^5,11^.

MINFLUX was also shown to track labeled proteins with record (sub)millisecond per nanometer spatiotemporal precision^9,12^. This finding brings up the question whether and to which extent MINSTED is suitable for molecular tracking. In this Brief Communication, we show that MINSTED attains a similar spatiotemporal resolution as MINFLUX. Moreover, we show that updating the position estimate of the emitter with each detected photon provides an elegant way of following the molecule’s motion directly, without additional computation. The power of MINSTED tracking is highlighted by revealing rarely observed leaps of the motor protein kinesin-1 when walking on microtubules, such as sudden protofilament switching and sidestepping.

**Fig. 1:**
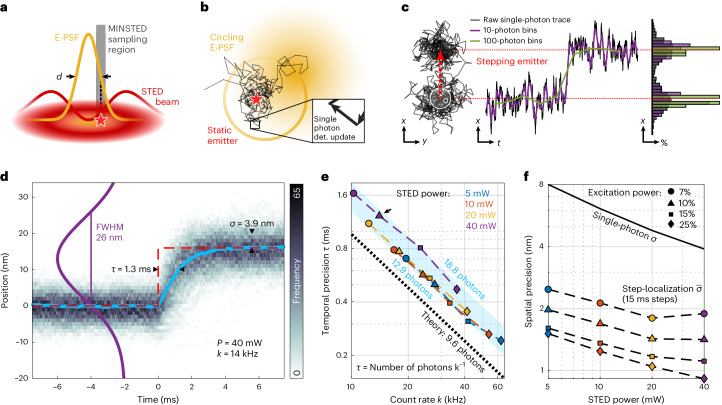
MINSTED localization principle and spatiotemporal precision. **a**, The STED E-PSF (orange) with FWHM *d* describes the emission probability of a fluorophore (red star) around the center of the donut-shaped STED-beam (red). In MINSTED, the fluorophore experiences the steep edge of the E-PSF (gray sampling region). **b**, MINSTED localizes by scanning the E-PSF (yellow) in circles around the assumed fluorophore position. Upon detection of each single photon (single photon det.), the circle center is shifted toward the detection position and by that moves (on average) toward the position of the fluorophore (star). **c**, MINSTED tracks a sudden jump in position (step) of a fluorophore with a few photon detections. Binning the circle centers (rendered for each detection) increases the visibility of the step while compromising temporal information (purple and green lines). Data in **a**–**c** are based on simulations using setup parameters. **d**, Overlaying *n* = 389 traces (histogram frequency shown as gray scale; STED power, *P* = 40 mW; E-PSF FWHM, 26 nm; count rate, *k* = 14 kHz) reveals a nearly exponential response function (blue) with a decay time *τ* = 1.3 ms and a single-photon spatial precision *σ* = 3.9 nm. **e**, Temporal precisions *τ* (relative standard errors <1% and thus not shown) as a function of the photon count rate $k$. The arrow indicates the data point representing the data of **d**. The blue shading indicates the span of minimal (12.9) and maximal (18.8) average number of photons needed for convergence to the new position. The ideal *τ* (theory) is shown as black dashed line. **f**, Single-photon precision and step-localization precision (relative standard errors <1‰ and thus not shown) as a function of STED beam power. Excitation powers are given in percentage of approximately 30 µW. The number of steps for each of the 16 conditions is given in Extended Data Table 2.

Like in STED microscopy, which has also been used to monitor one-dimensional protein movements by repetitive line-scan acquisition^13^, in single fluorophore localization by MINSTED the donut-shaped STED-beam reduces the space from which detected photons originate to subdiffraction dimensions. This space is described by the so-called effective point spread function (E-PSF, Fig. 1a) giving the normalized probability of the fluorophore to emit a photon. In our MINSTED implementation, the E-PSF is scanned on a circular trajectory around the assumed fluorophore position so that, upon each photon detection, the circle center is slightly shifted toward the assumed point of emission (Fig. 1b). At the same time, the STED intensity is increased and the circle diameter decreased. This reduces the full width at half maximum (FWHM) of the E-PSF, improving the positional information gain per photon, while keeping the emission rate and the exposure of the fluorophore to STED light constant. This procedure ultimately aligns the (average) circle center with the fluorophore position with continually increasing precision. Once the E-PSF is down-sized to a minimal FWHM *d*, each detected photon gives an update of the fluorophore position with subdiffraction precision (Fig. 1b). This single-photon-based localization update renders our MINSTED implementation particularly suitable for tracking. The circle center positions form a spatially and temporarily smeared-out track of the emitter motion (Fig. 1c). Note that an added benefit of STED is that the donut keeps the (fluorescence) background low due to its innate signal-suppressing capabilities, which is advantageous over MINFLUX in many applications.

To quantify the temporal and spatial uncertainties in MINSTED, we investigate the response of our method to an instantaneous fluorophore displacement. To measure this step response, we employed the fluorophore Cy3B on a MINSTED setup featuring beam wavelengths of 560 nm and 636 nm for excitation and STED, respectively. Rather than dislocating the fluorophore itself, which is technically difficult to execute sufficiently fast, we dislocated the co-aligned beams from a stationary fluorophore by step size *s* = 16 nm. The subsequent localization trace, describing the (sub)millisecond convergence of the beams toward the original position, revealed the step response. Averaging over multiple responses, taken every 15 ms, allowed us to extract the temporal precision *τ* as the decay constant of an exponential model and calculate the spatial precision as the standard deviation of the ensemble distribution (Fig. 1d).

Recording the responses for different excitation and STED powers revealed the *τ* ∝ *k*^−1^ scaling of the temporal precision (Fig. 1e). Translating the response time to an average number of photon detections *N*_C_ = *τk* showed that less than 20 photons were needed to converge to the emitter position. At a detection rate *k* = 62 kHz, MINSTED follows 16-nm steps with *τ* < 250 µs.

The single-photon spatial precision *σ* scales with the FWHM and reaches 4 nm at a maximum STED power of *P* = 40 mW. Averaging over the position estimates in between consecutive steps, further improves the spatial precision of the fluorophore position measurement. This step localization precision $\bar{\sigma }$ scales (besides scaling with the FWHM) with the number of the detections at a given step coordinate and hence with *k*^−0.5^. Averaging over a step of 15 ms yields $\bar{\sigma }$ < 1 nm for *k* = 36 kHz and *P* = 40 mW (Fig. 1f). For a fluorophore that carries out steps *s* ⪆ 2*σ*, the steps can be directly identified in the raw data. Therefore, when measuring discrete fluorophore steps with *s* ⪆ 2*σ*, both the temporal precision (*τ*), as well as the inter-step averaged spatial precision ($\bar{\sigma }$), which depends on the duration of the current step, can be gained simultaneously.

**Fig. 2:**
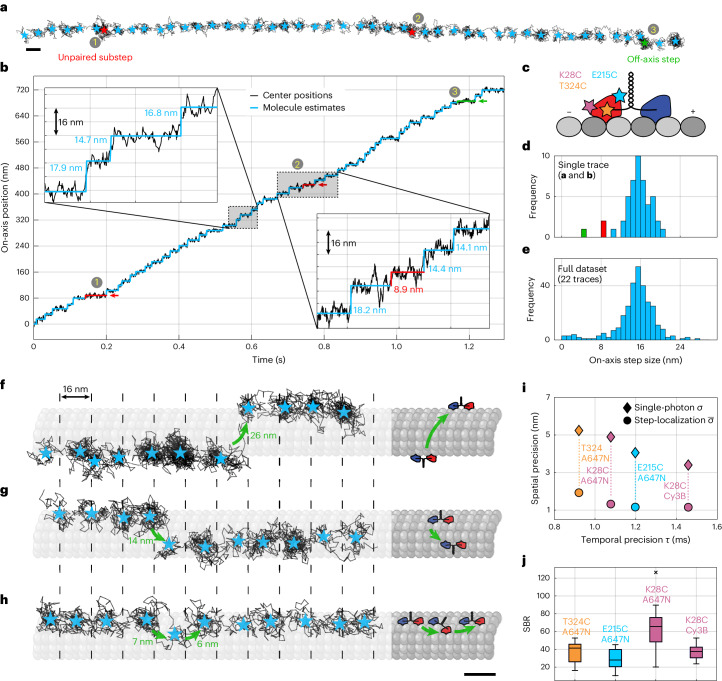
MINSTED tracking of kinesin-1. **a**, Stepping of kinesin-1 on a microtubule. Localizations (that is, circle center positions) are shown in black; light-blue stars indicate inter-step estimated fluorophore position. Red stars (1 and 2) indicate unpaired steps of ~8 nm along the microtubule axis. Green star (3) indicates mainly off-axis step. **b**, On-axis circle center position (black) and their inter-step average (light blue) of trace shown in **a**. **c**, Sketch of kinesin-1 homodimer and fluorophore position. **d**, Histogram of the on-axis step sizes of the trace displayed in **a** and **b** (*n* = 47 steps). **e**, Histogram showing on-axis step sizes from all 22 MINSTED traces of the same construct (*n* = 303 steps). **f**, MINSTED localization trace revealing a leftward 26 nm off-axis displacement while keeping its 16 nm stepping periodicity. **g**, Trace displaying a rightward 14 nm off-axis step associated with an on-axis ‘phase-shift’ of 8 nm. **h**, Back-and-forth off-axis stepping with 7 nm and 6 nm steps. The visualizations of the microtubule were created with BioRender.com. **i**, Median spatial and temporal precision of the MINSTED traces of the different kinesin-1 constructs (temporal and spatial standard errors <0.031 ms and <0.26 nm, respectively, and thus not shown). **j**, Boxplot of SBR for the different motor constructs. Each central marker indicates the median, the edges of the box indicate the 25th and 75th percentiles, and the whiskers span the range of data removed from outliers. Outliers are plotted as crosses; **i** and **j** are based on *n* = 5, 22, 26 and 18 traces of the constructs T324C-ATTO 647N, E215C-ATTO 647N, K28C-ATTO647N and K28C-Cy3B, respectively. Scale bars, 16 nm. Constructs, E215-ATTO 647N (**a**–**g**) and T324C-ATTO 647N (**h**).

To explore the power of MINSTED for protein tracking, we resolved the steps of the motor protein kinesin-1 walking on microtubules (Fig. 2a,b). Our tracking experiments were performed using an in vitro assay with three different constructs of kinesin-1 labeled with a single fluorophore (ATTO 647N or Cy3B) at different sites via maleimide coupling: in the front (E215C), in the rear (K28C) and in the middle (T324C) of the head, with respect to the motor’s walking direction (Fig. 2c).

With E215C-ATTO 647N, we recorded traces up to 736 nm in length with a temporal precision of <2 ms, displaying clearly identifiable steps in the raw data (Fig. 2a,b). The step-size histograms of the shown trace (Fig. 2d) and of the full E215-ATTO 647N dataset (Fig. 2e) both display a pronounced peak at the expected distance^9^ around 16 nm, corresponding to twice the distance between the successive binding sites of a kinesin head on the microtubule. Yet, we observe a subpopulation of steps with a size of ~8 nm. The shown trace also displays two ∼8 nm steps (Fig. 2a,b (1,2) marked in red). As they are preceded and succeeded by regular 16 nm steps, these steps could arise from the motor switching to a neighboring protofilament or from transiently detaching from the microtubule in order to enter a ‘slip state’^14^. In the latter, the motor reportedly re-engages with the same protofilament, but with the labeled and unlabeled heads in reverted order. However, as only one of the heads is tracked, the unpaired 8 nm step might also arise from kinesin constantly being attached to the same protofilament, but displaying an ‘inchworm’ step at this instant. A clearer case for the switching between protofilaments can nevertheless be made by the off-axis step (Fig. 2a,b (3) marked in green), which induces a shift of about 7 nm to the side, perpendicular to the assumed microtubule axis. This observation is best explained by kinesin-1 switching to another protofilament where it recovers its regular processivity^12,15^.

Pronounced protofilament switching is also displayed in other traces in Fig. 2f,g, where off-axis steps shift the straight stepping trajectories by up to 26 nm to the side. As the rate of detected photons remains constant during the step, we can nearly exclude that the steps after the shift arise from a different motor protein. A possible interpretation is that the motor interrupts its movement, detaches from the protofilament and re-engages with the microtubule on another protofilament. With such pronounced off-axis displacements, it is likely that the motor fully detaches from the protofilament and diffuses along the microtubule surface before re-engaging—occasionally even on the opposite side of the microtubule (Fig. 2f).

Closer inspection of all recorded traces revealed that protofilament switching occurs about every 65 steps (12 protofilament switches out of 779 total steps). Sideward displacements occur both to the right and to the left (Fig. 2f,g), in accordance with earlier observations made from tracking of kinesin-1 labeled with gold beads or quantum dots^15,16^. Furthermore, for about half of the cases, the ~16 nm periodicity was conserved, whereas, in the other half, the subsequent pattern of binding sites was shifted by ~8 nm after the switch. The apparent absence of a preferred binding site on the new protofilament supports the hypothesis that the motor is weakly bound to the microtubule when it switches protofilaments.

On rare occasions, kinesin-1 displayed sidestepping where it seemed to fleetingly switch to another protofilament before going back to the initial one. For example, a significant off-axis displacement of about 7 nm was observed during a regular plateau after a 16 nm on-axis step (Fig. 2h). With the subsequent regular step, the off-axis displacement was reversed, apparently bringing the labeled head back to the previous protofilament. Our microtubules were polymerized from highly pure tubulin with <1% of microtubule-associated protein and a minimal fraction of biotinylated (10%) and fluorophore-conjugated (2%, Alexa Fluor 488) tubulin. Roadblocks may arise from nonspecifically bound proteins (BSA-bt, pyranose oxidase and catalase) or inactive and nonfluorescent kinesins. The dwell time before a protofilament switch (median 20 ms) was not increased (median dwell time of all steps: 23 ms), unlike in a study with permanent roadblocks (~0.4 s)^15^. Thus, by using a minimally modified microtubule surface and by tracking the motor using a minimal label with MINSTED, we have observed protofilament switches of kinesin-1 that may be due to unknown roadblocks, local defects on microtubules, or switches that are actually intrinsic to the walking behavior of kinesin-1. The (detectable) detachments and reattachments occur with a probability of ~1.5%.

In a nutshell, using fluorescently labeled kinesin-1 as target, we have shown that MINSTED renders nanometer/millisecond spatiotemporal resolution (Fig. 2i) similarly to MINFLUX. The main advantage of MINSTED, however, is its ability to suppress background and thereby to improve the signal-to-background ratio (SBR) (Fig. 2j), resulting in an order of magnitude higher SBR than that achieved by MINFLUX (see comparison in Extended Data Table 4). This could make MINSTED more suitable for samples featuring high fluorescent background.

The deeply underlying reason why both MINSTED and MINFLUX localization require fewer detected photons is the same. Due to diffraction, defining a molecular coordinate with a diffracted light beam with high precision undeniably requires many photons. Whereas in conventional localization providing these photons is entirely up to the fluorophore, in MINFLUX and MINSTED the majority of the photons needed for localization is provided by the laser. Although this principle comes to full power in our MINSTED tracking, additional developments in fluorophore chemistry, the coordinate-finding algorithm, and the optical system are poised to improve the spatiotemporal resolution of coordinate-targeted fluorophore tracking even further.

## Methods

### MINSTED microscopes

We used two previously described MINSTED fluorescence microscopes^7,11^ with each a different pair of excitation (*λ* = 560 nm/635 nm) and STED wavelength (*λ* = 636 nm/775 nm). For the 635 nm STED system, the STED laser has been upgraded with a cladding-pumped double-clad fiber, amplifying the seed laser diode (Fiber: TB376, Le Verre Fluoré, Bruz, France; Pump Diodes: PLPT5 450KA, Osram). Both microscopes featured an electro-optic 2D scanning system (Conoptics) with a circling frequency of 125 kHz that positioned the co-aligned beam pairs with Angström precision in the focal plane. The lasers were pulsed with a repetition rate of 40 MHz/20 MHz repectively, while the excitation pulses and STED pulses had a duration of 0.1–0.2 ns and ≤1.5 ns, respectively. About 1 ns after each excitation pulse, a time gate for fluorescence detection with an avalanche photo diode was opened for 8 ns, to keep background low. In both setups the field-programmable gate array control modified the position of the co-aligned laser beam, as well as their power, in response to every detected photon. Lateral sample stability was ensured by tracking the position of metal colloids in the sample with back-scattered near-infrared light. A focus lock, tracking the reflection of an NIR beam from the coverslip–sample interface, stabilized the sample along the optical axis. The sample was translated and actively position-corrected with subnanometer precision by means of a three-axis piezo stage. The measurement control software was implemented in LabVIEW 2017 and MATLAB R2018b.

### MINSTED measurements

MINSTED was implemented using the previously described localization algorithm^7^. All measurements were executed with a circle diameter 2*r* matching the FWHM of the employed E-PSF. The center positions were updated upon each photon detection with a step size corresponding to *α* = 15% of the circling radius *r*. For the step temporal response measurements (Fig. 1d–f), the MINSTED localizations were initiated when four neighboring pixels in a confocal overview scan (1.6 ms dwell time, 80 nm pixel size) crossed an accumulated number of 40–80 counts. For kinesin measurements (Fig. 2) with Cy3B, the threshold was set to 4 (60 µs dwell time, 80 nm pixel size); while with ATTO 647N, it was chosen to 5 (60 µs dwell time and 50 nm/75 nm *x*/*y* pixel size). Localizations were terminated as soon as less then 16 photons were detected within a time *T*_t_. The STED powers, FWHMs, sampling radii and termination times *T*_t_ for all measurements carried out as a part of this study are presented in Extended Data Table 1.

In theory (and for a constant ratio of sampling radius *r* and FWHM), the STED power does not influence the photon detection rate during a MINSTED measurement. Hence, the time to respond to a small step should not depend on the STED power applied. Nonetheless, a higher STED power leads to a better spatial precision, since both the single-photon spatial precision σ and the localization precision $\bar{\sigma }$ scale linearly with the FWHM of the E-PSF. Thus, in theory, the highest possible STED power (minimum FWHM) would be the optimal choice to obtain both the ultimate spatial and temporal precision. Yet, in a real measurement, increasing the STED power decreases the photon detection rate at a constant *r*/FWHM ratio and excitation power (Fig. 1e). This is likely to be due to imperfections of the STED donut zero or due to transitions to fluorophore dark states. The scaling of the spatial precision with the STED power is as expected (Fig. 1f). Please note that the deviation of $\bar{\sigma }$ from a power law stems from the fact that the detection rate and thus the number of photons within a step plateau depends on the STED power. Choosing the STED power for a MINSTED tracking measurement is thus a compromise between either optimizing the single-photon spatial precision σ (high STED power) or aiming for a high detection rate to improve the temporal precision *τ* (low STED power). Since the stepping behavior of kinesin-1 at the given sample conditions leads to one hop of size 8 nm/16 nm per approximately 23 ms, which is a relatively small distance in a relatively long time (when comparing to the values in Fig. 1e,f), we chose relatively large STED power levels, that is, small FWHMs. This choice allowed us to attain a spatial precision that detected kinesin-1 steps from single photon data. The chosen STED power levels represent the maximum levels at which stable measurements can be performed (except for one dataset at 50 mW for K28C-ATTO 647N).

The choice of step-size parameter *α* = 0.15 is based on the following consideration: the response time *τ* scales with the step-size parameter as $\tau \propto \frac{1}{\alpha }$ (see ‘Analytical calculation of the temporal precision’ section). The influence of *α* on the single-photon spatial precision cannot be derived analytically. Yet, we can elucidate a number of basic relationships: for large *α* values, the center positions (position estimates) of the sampling circle are only weakly correlated, since each new center position largely depends on the exact photon emission time (which determines the direction of the update step). In other words, the new center position only weakly depends on the prior center position. On the other hand, a small value of *α* causes a large correlation between the center positions. The number of correlated photons in the raw-data trace is inversely proportional to the step-size parameter: ${N}_{\mathrm{c}}\propto \frac{1}{\alpha }$. Higher correlation (smaller *α*) leads to a narrower spatial distribution of circle center positions throughout the tracking measurement, that is, an improved single-photon spatial precision. The response time increases at smaller *α* (as the correlation length needs to be overcome to adapt to the new position). Therefore, the choice of *α* implies a compromise between temporal and spatial precision. The choice *α* = 0.15 is sufficiently small and allows the detection of the kinesin-1 jumps directly from the raw data.

In MINSTED measurements, the excitation and STED beams are scanned on a circular trajectory, to track the position of the fluorescent molecule. The question might therefore arise as to what extent the scanning process affects the measurement trajectory and introduces artefacts into the data. As mentioned above, the scanning was performed at a rate of 125 kHz (8 µs per circle), using electro-optic deflectors. The maximum photon detection rate in this study was about 60 kHz (Fig. 1e). For the kinesin measurements, the mean photon detection rates remained slightly below 15 kHz. Thus, about two to ten sampling circles were performed on average before a new photon was detected. Therefore, the emission of each photon can be considered as a stochastic event resulting from sampling the whole circle. For a scanning rate that is well below the photon detection rate, the tracking motion of the circle center would show circling artifacts (arising from the sampling trajectory). These artifacts would overlay the directed movement of the emitter. On the other hand, for an unrealistically high sampling rate on the order of the fluorescence lifetime (~1 ns^−1^), the stochastic temporal offset between excitation and photon detection would induce a large angular uncertainty regarding the position of excitation on the circular trajectory. Sampling/circling rates in between those extrema (with respect to the photon detection rate) should lead to traces that encode the motion of the emitter but do not contain artifacts arising from the sampling shape.

### Sample preparation

#### DNA origami

Ten microliters of gold nanorods (A12-40-980, Nanopartz) at 0.2 mg ml^−1^ in methanol were dried onto a coverslip, that was previously cleaned in an ultrasonic bath with Hellmanex II (Hellma), and then treated with an air plasma for approximately 15 min. The coverslip was glued to a microscope slide using double-sided Scotch tape, creating a flow channel. The latter was rinsed with phosphate-buffered saline (PBS) (1× PBS 7.4 pH) and then filled with 15 µl of 0.5 mg ml^−1^ biotinylated bovine serum albumin (BSA; A8649, Sigma-Aldrich) in PBS. After 4 min incubation and washing with 100 µl PBS, the channel was filled with 15 µl 0.5 mg ml^−1^ streptavidin (11721666001, Sigma-Aldrich) in PBS, and incubated for another 4 min. The channel was flushed with 100 µl of 10 mM MgCl_2_ in PBS before incubating 15 µl of the DNA origamis (3 × 3 6 nm, GattaQuant) for 15 min. Thereafter, the channel was washed with 400 µl of 75 mM MgCl_2_ in PBS and the 15 µl of the imager strand solution was added. The latter consisted of 5 nM of Cy3B coupled to the 3′ end of a DNA oligonucleotide (P1 sequence: 5′–3′ CTAGATGTAT, Metabion) in an oxygen-deprived reducing-oxidizing buffer^17^. Each 200 µl of this buffer consisted of 100 μl reducing-oxidizing buffer (10% (w/v) glycose, 12.5% (v/v) glycerol, 0.1 mM tris(2-carboxyethyl)phosphine (TCEP) and 1 mM ascorbic acid) and 100 μl PBS supplemented with 2 μl of oxygen removal enzyme mix (25 units of pyranose oxidase (P4234, Sigma-Aldrich) and 80 μl of catalase (C100, Sigma-Aldrich) with 170 μl of PBS), 1 μl of 200 mM methyl viologen dichloride hydrate (856177, Sigma-Aldrich) and 75 mM MgCl_2_.

### Kinesin

#### Expression constructs

Truncated human kinesin-1 (residues 1–560 with a C-terminal His-tag) with all solvent-exposed cysteines mutated to alanine or serine (C7S, C65A, C168A, C174S, C294A, C330S and C421A) and containing a unique cysteine residue for labeling at position E215C or T324C was expressed using plasmids K560CLM E215C^18^ (kindly provided by the Yildiz Lab, University of California, Berkeley) and K560CLM T324C^18^ (obtained from Addgene, #24460), respectively. A ‘cysteine-light’ truncated human kinesin-1 for labeling at K28C position (K560CLM K28C) was generated using QuikChange II (Agilent) site-directed mutagenesis method with CLM RP HTR plasmid^19^ obtained from Addgene (#24430) and used as a template. All constructs were verified by Sanger sequencing (Eurofins).

#### Protein expression and purification

The vectors were transformed into *Escherichia coli* BL21 CodonPlus(DE3)-RIL (Agilent). Cells were grown at 37 °C in LB medium supplemented with ampicillin (100 μg ml^−1^) and chloramphenicol (30 μg ml^−1^). At an optical density at 600 nm of 0.8–1.0, cells were transferred to 18 °C and expression was induced with 0.1 mM isopropyl β-d-1-thiogalactopyranoside. Cells were collected after overnight expression by centrifugation, frozen in liquid nitrogen and stored at −80 °C until further use. All subsequent purification steps were done at 4 °C.

The cell pellets were resuspended in lysis buffer (50 mM NaH_2_PO_4_ pH 8.0, 250 mM NaCl, 20 mM imidazole and 2 mM MgCl_2_) supplemented with complete ethylenediaminetetraacetic acid-free protease inhibitors (Roche), 20 μg ml^−1^ DNaseI, 10 mM β-mercaptoethanol and 1 mM ATP. The cells were lysed using a microfluidizer (Microfluidics) operated at a pressure of 0.9 MPa, and the lysates were clarified by centrifugation at 47,850*g* for 1 h at 4 °C. The cleared supernatants were loaded onto a HisTrap FF 5 ml (Cytiva) column pre-equilibrated with lysis buffer. The column was washed with 50 mM NaH_2_PO_4_ pH 6.0, 250 mM NaCl, 20 mM imidazole, 1 mM MgCl_2_, 10 mM β-mercaptoethanol and 0.1 mM ATP, and the protein was eluted with 50 mM NaH_2_PO_4_ pH 7.2, 250 mM NaCl, 500 mM imidazole, 1 mM MgCl_2_, 10 mM β-mercaptoethanol and 0.1 mM ATP.

Fractions containing kinesin were fivefold diluted with buffer A (25 mM PIPES pH 6.8, 2 mM MgCl_2_, 1 mM egtazic acid (EGTA), 0.2 mM TCEP and 0.1 mM ATP) before loading onto a HiTrapQ FF 5 ml (Cytiva) column pre-equilibrated with buffer A containing 100 mM NaCl. The column was washed with the same buffer, and the protein was subsequently eluted using a linear gradient of 100–1,000 mM NaCl in buffer A. The peak fractions containing kinesin were concentrated using Amicon Ultra (Merck Millipore) centrifugal units and further purified by gel filtration on a HiLoad 16/600 Superdex 200 pg (Cytiva) column equilibrated with buffer B (25 mM PIPES pH 6.8, 300 mM NaCl, 2 mM MgCl_2_, 1 mM EGTA, 0.2 mM TCEP and 0.1 mM ATP) to further improve the sample quality. Selected fractions containing kinesin were finally combined and concentrated, supplemented with 10% (w/v) sucrose, aliquoted, frozen in liquid nitrogen and stored at −80 °C. Purified proteins were analyzed by electrospray ionization mass spectrometry.

#### Labeling of kinesin

Kinesin was labeled with ATTO 647N maleimide (AD 647N-41, ATTO-TEC) or Cyanine3B (Cy3B) maleimide (19380, Lumiprobe) overnight at 4 °C. Excess dye was removed from the reaction mixture by size-exclusion chromatography (PD MiniTrap G-25, 28-9180-07, Cytiva) according to the manufacturer’s protocol. The degree of labeling was determined by ultraviolet–visible spectroscopy (DS-11+ Spectrophotometer, DeNovix) and mass spectrometry (ESI, maXis II ETD, Bruker). Sucrose was added to the labeled protein in a concentration of 10% (w/v), and aliquots were flash-frozen in liquid nitrogen and stored at −80 °C.

#### Preparation of microtubules

Biotinylated and fluorescently labeled microtubules were polymerized from 88% Cycled Tubulin (032005, PurSolutions, LLC), 10% Labeled Tubulin-Biotin-XX (033305, PurSolutions, LLC) and 2% Labeled Tubulin-Alexa Fluor 488 (048805, PurSolutions, LLC). The lyophilized tubulin variants were suspended in PEM80 buffer (80 mM PIPES, 0.5 mM EGTA and 2 mM MgCl_2_, pH 7.4) with 1 mM guanosine-5′-[(α,β)-methyleno]triphosphate (NU-405S, Jena Bioscience), and the solution was incubated for 30 min at 37 °C. Afterwards, the polymerized microtubules were centrifuged at 21,000*g* in a bench-top microcentrifuge (Fresco 21, Thermo Scientific) for 15 min, washed with PEM80 and centrifuged at 21,000*g* for 15 min. The microtubule pellet was resuspended in PEM80, aliquoted, flash-frozen in liquid nitrogen and stored at −80 °C.

#### Sample preparation

Flow chambers were constructed using oxygen-plasma-cleaned coverslips and double-sided adhesive tape. The chambers were incubated with 0.2 mg ml^−1^ biotinylated poly-l-lysine-polyethylene-glycol (PLL-PEG-bt) solution (PLL(20)-g[3.5]-PEG(2)/PEG(3.4)-biotin, Susos AG) supplemented with 1% (v/v) Tween 20 (P9416, Sigma-Aldrich) in ddH_2_O for 15 min, rinsed with PEM80, incubated with 10 μg ml^−1^ neutravidin (NVD; 31000, Thermo Fisher) in PEM80 for 5 min, and rinsed with PEM80. The flow chambers were incubated with microtubules diluted in 20 μM cabazitaxel (FC19621, Biosynth Carbosynth) in PEM80 for 5 min, rinsed with PEM80, blocked with 100 μg ml^−1^ biotinylated bovine serum albumin (BSA-bt; A8549-10MG, Sigma-Aldrich) in PEM80 with 20 μM paclitaxel (10-2095, Focus Biomolecules) added for 30 min, and rinsed with PM15 buffer (15 mM PIPES and 2 mM MgCl_2_, pH 7.4).

Labeled kinesin in measuring buffer (1 mM 1,4-dithiothreitol (6908.1, Carl Roth), 20 μM paclitaxel, 10 μg ml^−1^ BSA-bt, 1 mM methyl viologen, 1 mM ascorbic acid, 1 mM adenosine 5′-triphosphate (ATP; A3377-1G, Sigma-Aldrich) with an oxygen scavenger system (0.25 units pyranose oxidase (P4234, Sigma-Aldrich), 0.8 µl catalase from bovine liver (C100, Sigma-Aldrich) and 5% (w/v) d(+)-glucose (HN06.1, Carl Roth)) in PM15 buffer) was added and the flow chamber was sealed with picodent silicone putty or nail polish.

#### Analytical calculation of the temporal precision

During MINSTED tracking the center positions of the scan pattern is indicative of the position of the molecule with a standard deviation of *σ*_center_. In the case of an immobile emitter, this estimation is biased only by the positions of the previous detections. If the emitter moves, this estimation bias leads to a systematic offset of the position estimate toward the previous position of the molecule and several detections are needed to remove this bias. The number of detected photons or time needed to get to the unbiased situation can be calculated. At time *t* = 0 the molecule moves in the *x* direction by a step size *s* from *x* = 0 to *x* = *s*. A Gaussian E-PSF with the standard deviation *σ*_E_ is circled around the estimated fluorophore position at a radius *r* and the center is shifted by a fraction *α* of the radius toward the detection of fluorescent photons, which are detected at an average count rate of *k*.

The temporal derivative of the *x* coordinate is given by the product of the average detection rate *k*, the step size *αr* and the *x*-projected stepping probability *p*_*x*_(*x*(*t*)) divided by the total stepping probability *p*(*x*(*t*))

$$\dot{x}\left(t\right)=k\times \alpha r\times \frac{{p}_{x}\left(x\left(t\right)\right)}{p\left(x\left(t\right)\right)}.$$

The total stepping probability is given by integrating over the E-PSF along the sampling trajectory

$$p\left(x\left(t\right)\right)\propto \int \mathrm{{e}}^{-\frac{{\left(s-x\left(t\right)-r\cos \beta \right)}^{2}+{\left(r\sin \beta \right)}^{2}}{2{\sigma }_{E}^{2}}}{\mathrm{d}}\beta ,$$

where *β* is the angle that parameterizes the position of the E-PSF along its circular sampling trajectory, while the *x*-directed fraction is given by

$${p}_{x}\left(x\left(t\right)\right)\propto \int \cos \beta \times {\mathrm{e}}^{-\frac{{\left(s-x\left(t\right)-r\cos \beta \right)}^{2}+{\left(r\sin \beta \right)}^{2}}{2{\sigma }_{E}^{2}}}\mathrm{d}\beta .$$

Assuming an exponential behavior for the shape of the step response,

$$x\left(t\right)=s-s{\mathrm{e}}^{-\frac{t}{\tau }},$$

with a response time *τ*. Inserting into the differential equation and solving the integrals leaves us with

$$\frac{1}{\tau }{\mathrm{e}}^{-\frac{t}{\tau }}=\frac{k\alpha r}{s}\times \frac{{I}_{1}\left(\frac{{rs}{\mathrm{e}}^{-\frac{t}{\tau }}}{{\sigma }_{E}^{2}}\right)}{{I}_{0}\left(\frac{{rs}{\mathrm{e}}^{-\frac{t}{\tau }}}{{\sigma }_{E}^{2}}\right)},$$

where *I*_0_ and *I*_1_ represent Bessel functions of the first kind. If the step size $s$ is small compared to the radius of the scan pattern *r*, the step size can be assumed to approach zero. In this limit, the lifetime can be easily calculated

$$\tau =\frac{2{\sigma }_{E}^{2}}{{\alpha {kr}}^{2}\,}.$$

Within this time, a mean number of

$${N}_{{\mathrm{C}}}=\frac{{2\sigma }_{E}^{2}}{{\alpha r}^{2}}$$

photons is detected. As this is the number of photons that is needed to reduce a given center position offset to e^−1^, *N*_C_ can also be considered as a measure for the correlation length within the localization. The probability of a photon detection within the scan pattern and therefore the next center position depends on the current center position and therefore on the previous detections. Dependent on the scan parameters used, this correlation varies in length as described by *N*_C_.

Inserting the measurement parameter *α* = 0.15 as well as the relation $2r={{\mathrm{FWHM}}}=2\sqrt{2\mathrm{ln}\!\left(2\right)}{\sigma }_{E}$, we find *N*_C_ = 9.62 photons. The respective temporal response curve is shown in Fig. 1e.

#### Data analysis

The data analysis was performed in MATLAB R2021b.

#### Step temporal response (Fig. 1d–f)

The artificial stepping was controlled by the field-programmable gate array by modulating the driving voltage of the *x*-axis electro-optic scanner during each localization to perform a step of step size *s* = 16 nm, every *T* = 15 ms. After cutting off all circle center positions where the circle radius was not yet converged to its final value, the resulting traces with a duration of less than 31 ms or a number of photons less than 100 were discarded. Around each remaining step (see number of steps in Extended Data Table 2), we segmented an interval of [−*T*, *T*] (if possible). The circle center positions within each of those step intervals are denoted *X*(*t*) and *Y*(*t*) with a detection time *t* relative to the time of the respective step. In addition to the original data with irregular detection times, the steps were mapped onto a regular, common time interval $\widetilde{t}$ with a sampling time of *T*/5,000. The mapped positions $\widetilde{X}$ resulted from an interpolation algorithm, selecting the most recent spatial coordinate in *X* for each element in $\widetilde{t}$. After segmentation, all steps with a detection rate of less than 70% of the median value among all localizations and those featuring displacements of any $\widetilde{X}$ larger than (*s* + 2 std(*Y*)) were discarded.

To adapt for the global position offset of each step, the mean value *x*_0_ = 〈*X*(*t*)〉_*Z*_ was calculated within the time interval *Z* = (−10 ms < *t* < 0), and subtracted from the respective step responses $X$ and $\widetilde{X}$ (zeroing). Artificially adding the step size *s* to all $\widetilde{X}$ before the first photon arrival at all times $\widetilde{t}\ge 0$ resulted in a set of both temporally and spatially overlaid steps, jumping from a position *s* down to a position 0 at $\widetilde{t}=0$. The mean step response was calculated as an ensemble average of the interpolated responses $\widetilde{X}(\widetilde{t})$. The decay time ${\tau }_{0}$ was determined from the mean step response by fitting an exponential decay.

With this initial estimate of the response time, the interval *Z* was adapted to (−*T* + 5*τ*_0_ < *t* < 0), and the zeroing was repeated to ensure convergence of the trace for all times within *Z*. The final response time $\tau$ was estimated (now from the corrected positional values) as above.

For the estimation of the single-photon spatial precision *σ*, the standard deviation of $\widetilde{X}(\widetilde{t})$ among the measured steps was evaluated. The resulting *σ* was computed as the mean of those standard deviations over $\widetilde{t}$ for $5\tau < \widetilde{t} < T$. The step-localization spatial precision was estimated as $\bar{\sigma }=\frac{{\rm{std}}{\left({\left\langle X\left(t\right)\right\rangle }_{5\tau < t < T}\right)}_{{\rm{steps}}}}{\sqrt{2}}$. Assuming that all steps sample the same positional value (in the given time interval), the standard deviation over the temporal average would give the spatial precision of the mean position. Due to the statistical error in the zeroing step, and as the uncertainty of the latter can be assumed equal to the step-localization spatial precision, a factor of ${2}^{-1/2}$ is multiplied.

#### Kinesin stepping (Fig. 2)

The raw tracks of the kinesin movement were cut by removing the first center positions where the sampling radius had not yet converged to its minimal value as well as the last 16 photons (to remove potential random motion due to pure background detection after a bleaching event occurred). Thereafter, multiple filters were applied, to identify traces that display actual kinesin movement with at least a few steps. First, all traces with a duration shorter than *T*_min_ were discarded. We performed an initial rotation operation on the remaining traces, by forcing the first and last center position to lie on the *x* axis. We then estimated the covered distance from the minimal and maximal *x* deflection and filtered for minimal distances of *D*_min_. The single-photon spatial precision *σ* was approximated by the *y*-axis standard deviation. Traces with a single-photon spatial precision falling outside the interval [*σ*_min_, *σ*_max_], and traces with an aspect ratio std(*X*)/std(*Y*) smaller than *r*_min_ were discarded. The current count rate *k*(*t*) was estimated as the reciprocal of the moving mean of each 20 values over the time differences of consecutive photon detection times and was further smoothed by applying a moving mean of 50. Another filter was applied to the standard deviation of *k*(*t*) in order to discard traces with large fluctuations (>${\sigma }_{\max }^{k}$) in brightness. Such traces are likely to be disturbed by the presence of multiple emitters in the sampling, or events where one emitter was lost and a second one found after a short dark period. The filtering parameters for the recorded datasets are presented in Extended Data Table 3.

All remaining traces were fitted with a linear polynomial and rotated parallel to the *x* axis. On the rotated traces, we performed an edge detection, using the function findchangepts (MATLAB R2021b) in both *x* axis and *y* axis with the penalty parameter ‘MinThreshold’ set to 130(〈std(*Y*)〉_traces_)^2^ and the ‘Statistics’ parameter set to ‘mean’. Detected steps in the *y* axis were discarded if they referred to a detection index closer or equal to 20 photons with respect to an *x*-axis step. Finally, the mean positions between the detected steps were computed and those covering a Euclidian distance of ≤5 nm were discarded. Re-computing the mean coordinate between each two steps left us with an initial set of plateau positions. To retrace the local microtubule orientation for each single trace, we computed the angle of the stepping vectors between the plateau positions with respect to the *x* axis and rotated each trace so that the median angle vanished. This enforced an alignment of the major stepping direction with the *x* axis and mostly repressed an angular alignment with respect to off-axis stepping. With those final rotated traces, we repeated the edge detection described above.

The single-photon spatial precision $\sigma$ of the step plateaus was calculated as the standard deviation of the axial spatial coordinate from the mean plateau position. The step plateau localization precision $\bar{\sigma }$ was estimated as described in ref. ^7^ by calculating the standard deviation of the respective step plateau coordinates with moving means of increasing numbers $M$ applied. Modeling those values with the function ${\sigma }_{{\rm{est}}}\left(M\right)=a/{\left(b+M\right)}^{c}$ to find the parameters under the given constraints (*a* < 0, *b* < 0, 0 < *c* < 0.5) results in the step plateau localization precision $\bar{\sigma }={\sigma }_{{\rm{est}}}(M=N)$, where $N$ denotes the total number of position estimates along the step plateau. A detailed explanation is given in ref. ^7^.

The temporal precision was estimated by first translating each step to a time interval [−*T*, *T*] around the estimated stepping time (with *T* = 10 ms). We then transformed the spatial coordinate by mapping the mean pre-step plateau to zero and the mean post-step plateau to one. We then modeled the transformed step with the function

$$f\left(t\right)=\max\left[0;1-{\rm{e}}^{\left(-\frac{t-{t}_{0}}{\hat{\tau}}\right)}\right],$$

to fine-tune the step’s temporal offset ${t}_{0}$. After translating the step temporarily by this offset, we mapped it onto a common time interval [−*T*, *T*] with a regular temporal spacing of *T*/500 as already performed in the data analysis of the artificial steps. Steps with $\hat{\tau }\ge 5\;{\rm{ms}}$ were discarded at this stage of the analysis. Such large decay times were probably due to off-axis stepping or multiple steps in quick succession. We then overlayed all remaining steps within each of the four datasets by computing the median position for all times. This averaged step was then flipped and fitted with ${\mathrm{e}}^{\left(-\frac{t}{\tau}\right)}$ for times *t* ≥ 0, resulting in the final estimate for the temporal precision $\tau$.

The SBR was estimated by extracting a mean background *k*_b_ rate for each dataset, while the overall detection rate $k$ was given for each trace by the total number of detections divided by the time interval of each localization. The background rate ${k}_{{\mathrm{b}}}$ was estimated by counting the number of photons from the end of each trace that were detected with > *T*_t_/16 (*T*_t_ is the termination time; see above for explanation) time difference with respect to the previous photon—that is, at a rate below the termination rate. Summing over all of those so-called background photons and dividing by the sum of all termination times, resulted in the average background rate ${k}_{{\mathrm{b}}}$ for the respective dataset. For this analysis, we did not only use the filtered traces but all recorded localizations. The SBR was then computed as (*k* − *k*_b_)/*k*_b_.

### Reporting summary

Further information on research design is available in the Nature Portfolio Reporting Summary linked to this article.

## Online content

Any methods, additional references, Nature Portfolio reporting summaries, source data, extended data, supplementary information, acknowledgements, peer review information; details of author contributions and competing interests; and statements of data and code availability are available at 10.1038/s41592-024-02209-6.

### Supplementary information

Reporting Summary

## Figures and Tables

**Extended Data Table 1:**
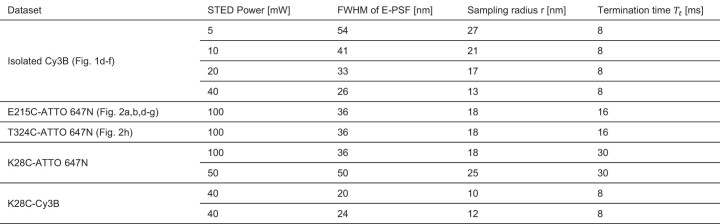
MINSTED measurement parameters.

MINSTED parameters used in the measurements of the DNA origami sample (Fig. 1d–f, isolated Cy3B) and the kinesin-1 constructs (Fig. 2).

**Extended Data Table 2:**
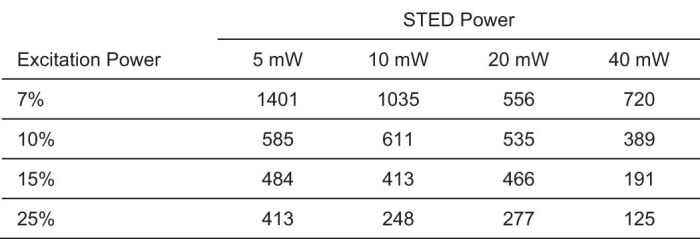
Number of steps in DNA origami measurements after filtering.

Post-filtering number of steps for each four different power conditions of excitation and STED light in the DNA origami measurements (Fig. 1d–f). The excitation powers are given in percentage of approx. 30 µW. All measurements were performed on the same DNA origami sample.

**Extended Data Table 3:**
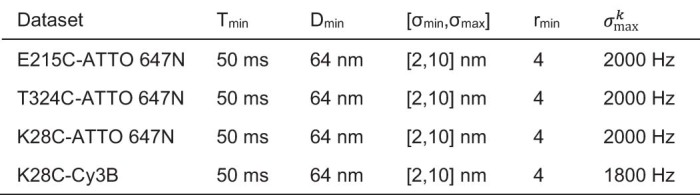
Filtering parameters applied to kinesin-1 datasets.

The data from the four kinesin-1 constructs was filtered with respect to the following parameters: the minimum trace duration T_min_, the minimum covered distance D_min_, the minimum and maximum off-axis spatial standard deviation [σ_min_,σ_max_], the minimum ratio of spatial standard deviations in on- and off-axis direction r_min_ and the maximum brightness (detection rate) fluctuations ${\sigma }_{\max }^{k}$.

**Extended Data Table 4:**
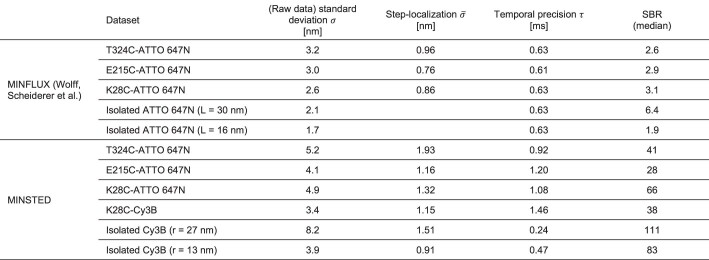
Current MINSTED and MINFLUX tracking performance.

The spatial and temporal precision as well as the SBR of the MINFLUX tracking data from ref. ^9^ and the MINSTED data from the present study are displayed for comparison.

## Data Availability

The relevant data is available at ref. ^20^.

## Code availability

The custom analysis codes are available at ref. ^20^.
